# Evaluation of odonto/osteogenic differentiation potential from different regions derived dental tissue stem cells and effect of 17β-estradiol on efficiency

**DOI:** 10.1186/s12903-020-01366-2

**Published:** 2021-01-07

**Authors:** Young-Bum Son, Young-Hoon Kang, Hyeon-Jeong Lee, Si-Jung Jang, Dinesh Bharti, Sung-Lim Lee, Byeong-Gyun Jeon, Bong-Wook Park, Gyu-Jin Rho

**Affiliations:** 1 Department of Theriogenology and Biotechnology, College of Veterinary Medicine and Research Institute of Life Science, Gyeongsang National University, 501 Jinju-daero, Jinju, GN 660-701 Republic of Korea; 2 Department of Oral and Maxillofacial Surgery, Changwon Gyeongsang National University Hospital, Changwon, Republic of Korea; 3 Department of Oral and Maxillofacial Surgery, Gyeongsang National University School of Medicine, Jinju, Republic of Korea; 4 Department of Biology Education, Gyeongsang National University, Jinju, Republic of Korea; 5 Department of Dentistry, Hanil Hospital, Jinju, Republic of Korea

**Keywords:** Odonto/osteoblast, Mesenchymal stem cells, Dental tissue, Dental pulp stem cells, 17ß-estradiol

## Abstract

**Background:**

The dentin is a tissue, which is formed by odontoblasts at the pulp interface of the teeth that supports the enamel. Odontoblasts, the cranial neural crest cells are derived from ectodermal mesenchymal stem cells (MSCs) and are long and polarized cells. They are present at the outer surface of dentin and play a prominent role about dentin formation. Recently, attention has been focused on induction of odontoblast using various type of MSCs and effects of the 17ß-estradiol supplementation. In this study, we establish an efficient odonto/osteoblast differentiation protocol using 17ß-estradiol supplementation while comparing the odonto/osteoblast ability of various dental MSCs.

**Methods:**

Same donor derived four types of dental MSCs namely dental pulp stem cells (DPSCs), stem cells from apical papilla (SCAP), dental follicle stem cells (DFSCs), and periodontal ligament stem cells (PDLSCs) were evaluated for their stemness characteristics and potency towards odonto/osteoblast (Induced odonto/osteoblast) differentiation. Then 17ß-estradiol supplementation of 0 and 10 µM was applied to the odonto/osteoblast differentiation media for 14 days respectively. Furthermore, mRNA and protein levels of odonto/osteoblast markers were evaluated.

**Results:**

All of the experimental groups displayed stemness characteristics by showing adipocyte and chondrocyte differentiation abilities, expression for cell surface markers and cell proliferation capacity without any significant differences. Moreover, all dental derived MSCs were shown to have odonto/osteoblast differentiation ability when cultured under specific conditions and also showed positive expression for odontoblast markers at both mRNA and protein level. Among all, DPSCs revealed the higher differentiation potential than other dental MSCs. Furthermore, odonto/osteoblast differentiation potential was enhanced by supplementing the differentiation media with 17ß-estradiol (E2).

**Conclusions:**

Thus, DPSCs possess higher odonto/osteogenic potential than the SCAPs, DFSCs, PDLSCs and their differentiation capacity can by further enhanced under E2 supplementation.

## Background

Dentin is a tissue that supports enamel and occupies most of the teeth. It is composed of mineral substance and calcified tissues. The caries, periodontal diseases and physical injuries, are the most frequently occurring tooth problems that cause dentin loss. In this process, odontoblasts are destroyed and hence induction of odontoblast-like cell from stem cells may prove helpful [1–3]. Odontoblast differentiation is important to the regeneration of dentin [4]. Therefore, many studies have focused on odontoblast differentiation using stem cells.

The dentin and bone are composed of minerals such as calcium and phosphorus, and most of them are composed of collagen type I organic substance. In addition, both tissues are hard and calcified, and they also express similar proteins such as osteonectin (ON), osteocalcin (OCN), osteopontin (OPN), alkaline phosphatase (ALP), dentin sialophosphoprotein (DSPP) and dentin matrixprotein 1 (DMP-1) [5, 6, 8–11]. As described previously, odontoblasts express ALP, DMP-1 and DSPP, which are the main factors for mineralization [5, 6, 8–13]. The DMP-1 and DSPP belongs to the integrin-binding ligand N-linked glycoprotein family [12]. These two proteins play crucial roles in the formation of mineralized tissues and are expressed at high levels in dentin and at very low levels in long bones [12, 13].

MSCs have the ability to self-renew, possess immunomodulatory properties and potency to differentiate into multi-lineages including odonto/osteoblasts under specific induction conditions [6–10]. Accordingly, there are increasing interests in enhancing the efficiency of differentiation into odonto/osteoblasts for pulp regeneration. Therefore, selection of highly suitable stem cell sources and efficient induction methods into odonto/osteoblast are highly important so that MSCs can be used as therapeutic agents for regenerative medicine.

Studies have been carried out on odonto/osteoblast differentiation using various tissue derived MSCs. Odonto/osteoblast differentiation was conducted using human bone marrow MSCs (BM-MSCs) [8]. However, isolation of BM-MSCs needs invasive procedure, prone to cell contamination, have low cell isolation number and their potency is donor age-dependent. MSCs from umbilical cord matrix (UCM) and umbilical cord blood (UCB) are good candidates for odontoblast differentiation [6, 7]. UCM and UCB derived MSCs are known to have high stemness properties, but they cannot be used without tissue storage at birth. Recently, MSCs from dental tissue have been evaluated for the presence of higher odonto/osteoblast differentiation potential. Dental tissues have gained more attention since MSCs can be isolated without invasive procedures from extracted tooth, which is considered a biomedical waste in dental care. In addition, many studies performed odonto/osteoblast differentiation using dental-derived MSCs [10, 11, 14]. However, the comparative evaluation of different regions of single donor derived dental MSCs have not been performed for the odonto/osteoblast differentiation.

Researchers have also utilized hormones as differentiation inducers. One such example includes estrogen hormone that regulates cell proliferation and differentiation [15, 16]. Interestingly, 17ß-estradiol (E2)-supplementation has been found to enhance the odonto/osteoblast differentiation capacity of MSCs by regulating estrogen receptor-α (ER-α), c-Src, and mitogen-activated protein kinases (MAPKs) [15]. E2 supplementation led to improved odonto/osteoblast differentiation in MSCs, as evidenced by an increased expression of odontoblast markers and mineral matrix formation [15, 16]. However, extensive studies on the effect of E2 supplementation on calcium content evaluation are still lacking. Keeping these necessities in mind, differentiation-promoting effect of E2 supplementation was also evaluated.

Therefore, present study primarily focused on deriving odonto/osteoblast differentiation by targeting two important themes. First target was to select the highly efficient dental tissue derived cell region (among four different kinds of dental stem cells derived from dental pulp (DPSCs), follicle (DFSCs), root apical papilla (SCAPs) and periodontal ligament (PDLSCs) on the basis of comparative evolution of all parts derived dental MSCs for their ability to undergo odonto/osteoblast differentiation under same culturing conditions. Whereas, second target focused on further evaluating the effect of 17ß-estradiol supplementation to enhance the odonto/osteoblast differentiation ability of selected region.

## Methods

### Chemicals and media

All chemicals were purchased from Sigma (St. Louis, MO, USA) and media from Gibco (Gibco Life Technologies, Gaithersburg, MD, USA), unless otherwise specified.

### Culture of dental pulp-derived mesenchymal stem cells and odonto/osteoblasts

Patients provided informed consent for the collection of dental tissues, in accordance with the requirements of the Ethics Committee of Gyeongsang National University Hospital (GNUH-IRB-2018-11-002-001). Dental follicle, pulp, apical papilla, and periodontal ligament were collected from the extracted immature wisdom teeth of eight patients (four men and four women) with an average age of 18.5 ± 2.3 years. MSCs were isolated from the dental tissues as previously described [17–19]. In brief, each tissue sample was washed twice with Dulbecco’s phosphate-buffered saline (DPBS) containing 1% penicillin/streptomycin (10,000 IU and 10,000 µg/mL, respectively). After washing, the samples were cut into 1–3 mm^2^ explants, digested by incubating in DPBS containing collagenase type I (1 mg/mL) at 37 °C for 40 min and pipetted at 15 min intervals. To prevent additional digestion, digested tissue samples were washed with Advanced Dulbecco’s modified Eagle’s medium (ADMEM) containing 10% fetal bovine serum (FBS) and filtered through 100 µm and 40 µm nylon cell strainers (Falcon®, Franklin, NJ, USA) to harvest single-cell suspensions. The cell pellets were centrifuged at 500 × *g* for 5 min and placed in ADMEM supplemented with 10% FBS at 37 °C, in a humidified incubator at 5% CO_2_. The culture medium was changed every three days, and colonies formed from single cells were dissociated with 0.25% trypsin EDTA solution and sub-cultured. Isolation of the odontoblasts was performed following previously described methods with minor modifications [20]. Briefly, the teeth were cut at the cement-enamel junction, harvested dental pulp chamber, and washed with DPBS. After being digested in a mixture of collagenase type I (1 mg/mL) and hyaluronidase (0.076 mg/mL) at 37 °C for 30 min, the pulp chamber was mechanically dissociated with pipetting and filtered through 100 µm and 40 µm nylon cell strainers, then centrifuged at 500×*g* for 3 min. The cell pellet was cultured on poly-l-lysine coated slides (Thermo Fisher Scientific, Waltham, MA, USA) with Opti-MEM reduced serum medium (Gibco, Paisley, UK), which was changed every three days.

### Flow cytometry

MSCs were evaluated for the expression of stem cell specific positive (CD44, CD73, CD90 and CD 105) and negative (CD34 and CD45) cell surface markers using BD FACSVerse™ instrument (BD biosciences, Franklin Lakes, NJ, USA) (Table 1) [17]. Briefly, MSCs (1 × 10^5^) were stained with fluorescein isothiocyanate (FITC)-conjugated anti CD34 (mouse monoclonal, BD Biosciences, Franklin Lakes, NJ, USA), CD45 (mouse monoclonal, BD Biosciences, Franklin Lakes, NJ, USA), CD44 (mouse monoclonal, BD Biosciences, Franklin Lakes, NJ, USA), CD73 (mouse monoclonal, BD Biosciences, Franklin Lakes, NJ, USA), and CD90 (mouse monoclonal, BD Biosciences, Franklin Lakes, NJ, USA) for 1 h. Analysis of CD105 (mouse monoclonal, Santa Cruz Biotechnology, Dallas, TX, USA) protein expression was performed by treating the cells with primary antibody for 1 h followed by staining with FITC-conjugated secondary antibody (Santa Cruz Biotechnology, Dallas, TX, USA). To remove the unbounded antibodies, cells were washed with DPBS. Finally, stained cell pellet was then suspended in 500 µL DPBS and measured by flow cytometry. All antibodies were diluted 1:100 with 1% bovine serum albumin (BSA). The standard was established by FITC mouse IgG, isotype control. (BD Biosciences, Franklin Lakes, NJ, USA).

**Table 1:** Lists of primers used in RT-qPCR analysis

Gene name (symbol)	Primers sequence	Product size (bp)	Anneal. temp (°C)
Fatty acid-binding protein4 (FABP4)	F: TGAGATTTCCTTCATACTGG R: TGGTTGATTTTCCATCCCAT	128	60
Lipoprotein lipase (LPL)	F: AGACACAGCTGAGGACACTT R: GCACCCAACTCTCATACATT	137	60
Peroxisome proliferator-activated receptor (PPARγ)	F: TTGCTGTCATTATTCTCAGT R: GAGGACTCAGGGTGGTTCAG	124	60
Aggrecan (ACAN)	F: AGTGGATTGGCTTGAACGAC R: AGTGGCGAAGAAGTTGTCAG	113	60
Collagen, type X, alpha 1(COL10A1)	F: GCAAACATGCTGCCACAAAC R: GATGAAGAACTGTGCCTTGGTG	141	60
Alkaline phosphatases (ALP)	F- AGCTCATCTCCAACATGGAC R- ACCTTGGCTGTAGTCATCTG	354	60
Dentin matrix acidic phosphoprotein 1 (DMP-1)	F- AGAGAGAGATGGGAGAGCTGCGC R- TGACCTTCCATCTGCCTCCTGTTC	128	60
dentin sialophosphoprotein (DSPP)	F- TCCTTTTGAAGCCTTTTAAGCCATT R- TGGTTTGCTTTGAGGAACTGGAAT	117	60
Estrogen receptor alpha (ER-α)	F- TGGGCTTACTGACCAACCTG R- CCTGATCATGGAGGGTCAAA	115	60
BCL2-associated X protein (BAX)	F: TGCTTCAGGGTTTCATCCAG R: GGCGGCAATCATCCTCTG	170	60
BCL-2 antagonist/killer 1 (BAK)	F: GGCACCTCAACATTGCATGG R: CAGTCTCTTGCCTCCCCAAG	144	60
B-cell CLL/lymphoma 2 (BCL2)	F: GGCTGGGATGCCTTTGTG R: CAGCCAGGAGAAATCAAACAGA	66	60
Baculoviral inhibitor of apoptosis repeat-containing 5 (BIRC)	F: CAGATGACGACCCCATGCAA R: GAGGCCTCAATCCATGGCAG	112	60
Tyrosine 3-monooxygenase/tryptophan 5-monooxygenase activation protein, zeta (YWHAZ)	F: CGAAGCTGAAGCAGGAGAAG R: TTTGTGGGACAGCATGGATG	113	60

### Multi-lineage differentiation

Dental-derived MSCs were differentiated at passage four into adipocytes, and chondrocytes using suitable culture conditions as previously described [18, 19]. For adipocyte differentiation, cells were cultured in DMEM containing 10% FBS, 100 µM indomethacin, 10 µM insulin, and 1 µM dexamethasone for 21 days. The accumulation of lipid droplets was evaluated by Oil Red O staining. For chondrogenesis, cells were cultured in StemPro chondrocyte differentiation basal medium and 10% StemPro chondrogenesis supplement (Invitrogen, Carlsbad, CA, USA) for 21 days. The deposition of proteoglycans was confirmed by Alcian blue staining.

### Cell proliferation

To evaluate the proliferation of DPSCs, SCAPs, DFSCs and PDLSCs, the population of doubling time (PDT) was performed [21]. All MSCs were seeded into 6-well plates. After every 72 h, the cells were detached and counted using hematocytometer. The PDT of the MSCs was calculated according to the formula, PDT = log2 + T/(logNH − log NI), where T is culture time, logNH is harvest cell number and log NI is initial cell number.

### In vitro differentiation into odonto/osteoblasts

Dental-derived MSCs were cultured in ADMEM supplemented with 10% FBS and differentiated at passage four into odonto/osteoblasts as previously described [1, 22, 23]. To investigate their capacity to differentiate into odonto/osteoblasts, all the four types of dental MSCs (at 70 ~ 80% confluence) were cultured in DMEM supplemented with 10% FBS, 1% penicillin/streptomycin, 10 mM ß-glycerophosphate, 50 µg/mL ascorbate-2-phosphate, 10 nM dexamethasone for 14 days. The medium was changed every two days [15]. Afterwards, DPSCs were cultured in DMEM supplemented with 10% FBS, 1% penicillin/streptomycin, 10 mM ß-glycerophosphate, 50 µg/mL ascorbate-2-phosphate, 10 nM dexamethasone with 10 µM E2 supplementation for 14 days [15]. Differentiated cells were then fixed with 4% paraformaldehyde, and stained with 5% silver nitrate solution (Von kossa staining) and ALP staining was performed by Western Blue® Stabilized Substrate (Promega, USA) following manufacturer’s instructions.

### Real-time quantitative polymerase chain reaction (RT-qPCR)

Total RNA was extracted from the MSC control group and induced odontoblasts using the easy-spin total RNA Extraction Kit (iNtRON, Seongnam, Korea), and quantified using a Nanodrop 1000 spectrophotometer (Thermo Fisher Scientific, Waltham, MA, USA). Complementary DNA (cDNA) was synthesized from total purified RNA (2 μg) using HisenScript RT PreMix kit (iNtRON, Seongnam, Korea) with 10 μM OligodT primer at 42 °C for 50 min. cDNA samples were diluted to a uniform concentration of 50 ng/μl. The RT-qPCR was performed using a Rotor-Gene Q cycler (Qiagen, Hilden, Germany) and RealMOD™ Green AP 5 × qPCR mix (iNtRON, Seongnam, Korea) containing 200 nM of forward and reverse primers (Table 2). RT-qPCR setting included denaturation at 95 °C for 60 s followed by 50 cycles of 95 °C for 10 min, 60 °C for 6 s, and 72 °C for 4 s. Gene expression was normalized to the mRNA levels of a control gene, Tyrosine 3-monooxygenase/tryptophan 5-monooxygenase activation protein, zeta polypeptide (YWHAZ).

**Table 2:** Lists of antibodies and their amount in immunocytochemistry analysis

Antibody	Company	Amount
FITC mouse IgG, isotype control	BD Pharmingen™	0.5 mg/ml
FITC mouse anti-human CD34	BD Pharmingen™	0.5 mg/ml
FITC mouse anti-human CD45	BD Pharmingen™	0.5 mg/ml
FITC rat anti-human CD44	BD Pharmingen™	0.5 mg/ml
Mouse anti-human CD73	BD Pharmingen™	0.5 mg/ml
FITC mouse anti-human CD90	BD Pharmingen™	0.5 mg/ml
Mouse monoclonal CD105	Santa Cruz biotechnology	200 µg/ml
FITC Goat anti-mouse IgG	Santa Cruz biotechnology	0.5 mg/ml

### Immunofluorescent staining

Immunocytochemical staining was performed as previously described [19]. Briefly, cells were fixed in 4% formaldehyde for 1 h and then blocked with 5% BSA diluted with DPBS for 1 h, and treated with primary antibodies (mouse monoclonal, Santa Cruz Biotechnology, Dallas, TX, USA), including ALP, DMP1, and DSPP for 1 h. Furthermore, cells were incubated with FITC-conjugated secondary antibodies at 20 °C for 1 h. All antibodies were diluted in a ratio of 1:100 with 1% bovine serum albumin (BSA). Counterstaining was performed with 1 µg/mL 4′,6-diamidino-2-phenylindole (DAPI) for 5 min to confirm the presence of nuclei. Fluorescence images were observed under fluorescence microscope (Leica, Wetzlar, Germany) and the number of odonto/osteoblasts were determined using Photoshop CS6 software by counting ALP, DMP-1 and DSPP positive cells in ten randomly captured high power fields.

### Alkaline phosphatase activity

Alkaline phosphatase activity of odonto/osteoblasts was measured using a TRACP and ALP assay kit (Takara Bio Inc, Noji higashi, Japan) according to the manufacturer’s instructions. Briefly, odonto/osteoblast differentiation of DPSCs was performed using 96-well plates (Nunc, NY, USA) for 14 d. The cells were then washed with DPBS and 50 µL of extraction solution and substrate solution were added to each well at 37 °C. The reaction was terminated using 50 µL of stop solution (0.5 N NaOH) and absorbance was determined at 405 nm by VERSAmax microplate reader (Molecular Devices, San Jose, CA, USA). All cellular proteins were measured using the BCA protein assay kit (Thermo Fisher Scientific, Waltham, MA, USA). ALP activity values were calculated as IU/mg to the total cellular protein.

### Calcium colorimetric assay

Calcium content was measured using a calcium colorimetric assay kit (Biovision, Milpitas, San Francisco, USA) according to the manufacturer’s instructions. In brief, 2 × 10^6^ DPSC-induced odonto/osteoblasts were suspended in a 500 µL calcium assay buffer and stored on ice, followed by pipette-based homogenization and centrifugation (500×*g*) for 5 min. The resulting supernatant was added to each well of 96-well plates and gently pipetted with 60 µL of calcium assay buffer and 90 µL of chromogenic reagent at 20 °C for 10 min. Absorbance at 575 nm was determined by VERSAmax microplate reader (Molecular Devices, San Jose, CA, USA). All cellular proteins were measured using the BCA protein assay kit (Thermo Fisher Scientific, Waltham, MA, USA). Calcium values were calculated as µg/mg to the total cellular protein.

### Statistical analysis

Statistical analyses were performed by one-way analysis of variance (ANOVA) using SPSS version 23 (IBM), and Tukey’s test was conducted for between-group comparisons. Data were represented as mean ± standard deviation (SD) and *p* < 0.05 was considered significant.

## Results

### Isolation and characterization of MSCs derived from dental pulp, follicle, apical papilla, and periodontal ligament

**Fig. 1:**
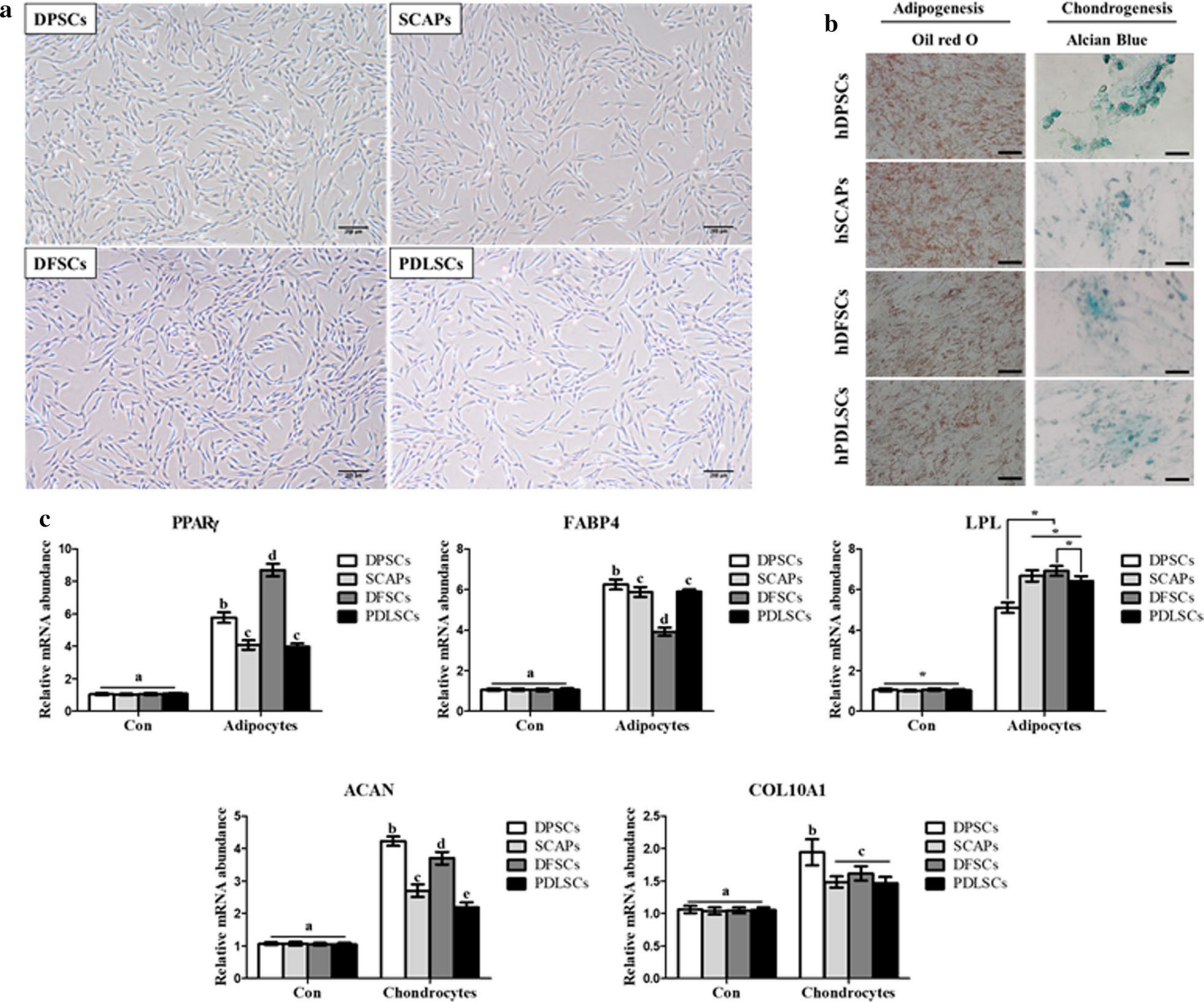
Characterization of stem cells from four types of dental tissue. Scale bar = 100 µm. **a** Cell morphology was observed at passage three by phase contrast microscope. The fibroblast-like dish-adherent growth cell morphologies were observed in all dental stem cells. **b** All dental stem cells were successfully differentiated into mesenchymal lineages, including adipocytes, and chondrocytes, which were detected by lineage-specific cytochemical staining: Oil red O for adipocytes, and alcian blue for chondrocytes. **c** The gene expression of lineage specific markers in differentiated adipocytes and chondrocytes in RT-qPCR. Data are represented by the mean ± SD of four independent experiments. Lettered subscripts indicate statistical differences between groups (*p* < 0.05)

**Fig. 2:**
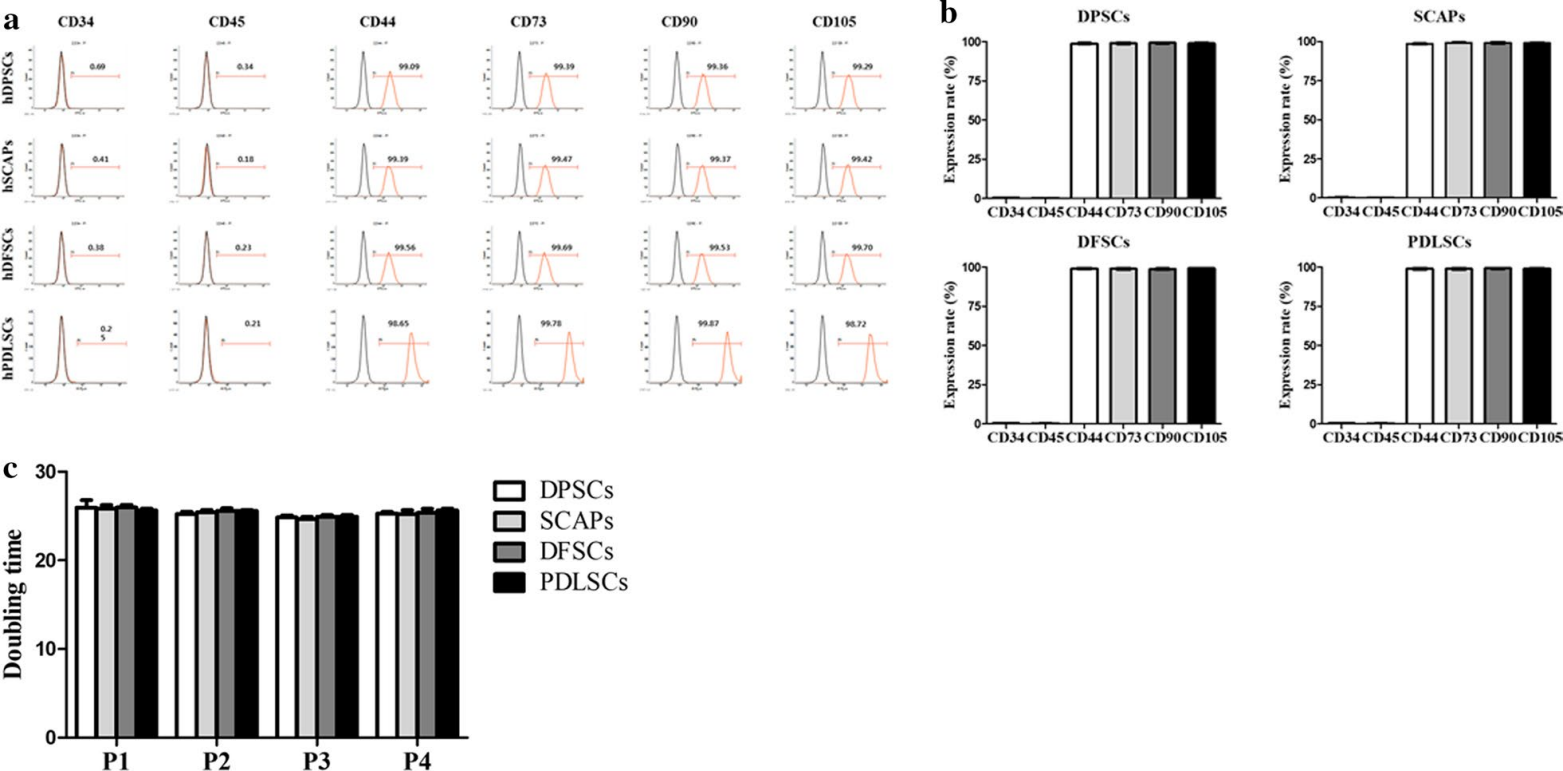
Cell surface makers and proliferation capacity in dental MSCs. **a**, **b** Flow cytometry revealed that the mesenchymal markers (CD44, CD73, CD90, and CD105) were positively expressed and hematopoietic markers (CD34 and CD45) were negatively expressed in all dental stem cells. **c** Population doubling time (PDT) of four kinds of dental mesenchymal stem cells. There was no difference during the culturing of cells. The values were expressed as mean ± SD of four independent experiments

Dental-derived MSCs (DPSCs, SCAP, DFSCs, and PDLSCs) were isolated and cultured. After passage two, the cells showed homogenous plate-adherent and fibroblast-like cell morphology (Fig. 1a). The in vitro differentiation potentials of DPSCs, SCAP, DFSCs, and PDLSCs at passage three were evaluated by differentiating each cell source into adipocytes, and chondrocytes. To evaluate successful adipocyte differentiation, accumulation of intracellular lipid droplets was confirmed by Oil red O staining (Fig. 1b). Adipogenic specific markers i.e. peroxisome proliferator-activated receptor (*PPARγ*), fatty acid-binding protein4 (*FABP4*) and lipoprotein lipase (*LPL*) were evaluated by RT-qPCR and revealed significantly higher (*p* < 0.05) expression as compared to non-differentiated MSCs (Fig. 1c). The accumulation of proteoglycans in differentiated chondrocytes was evaluated by Alcian blue staining and the expression of chondrocyte related genes i.e. aggrecan (*ACAN*) and collagen, type X, alpha 1 (*COL10A1*) were showed significantly (*p* < 0.05) higher in differentiated chondrocytes as compared to undifferentiated MSCs by RT-qPCR (Fig. 1b, c). Analyses of cell surface marker expression showed that all dental stem cells positively expressed MSCs specific markers (CD44, CD73, CD90, and CD 105; ≥ 97–99% of positive cells) and negatively expressed hematopoietic markers (CD34 and CD45; ≤ 2% of positive cells) (Fig. 2a). There was no difference in the cell morphologies and CD marker profiling among DPSCs, SCAP, DFSCs, and PDLSCs (Fig. 2b). The entire MSCs proliferation rate was analyzed in passages 1, 2, 3 and 4. The differences in PDT among the cells were not statistically significant (Fig. 2c).

### Odonto/osteogenic differentiation potential of DPSCs, SCAP, DFSCs, and PDLSCs

**Fig. 3:**
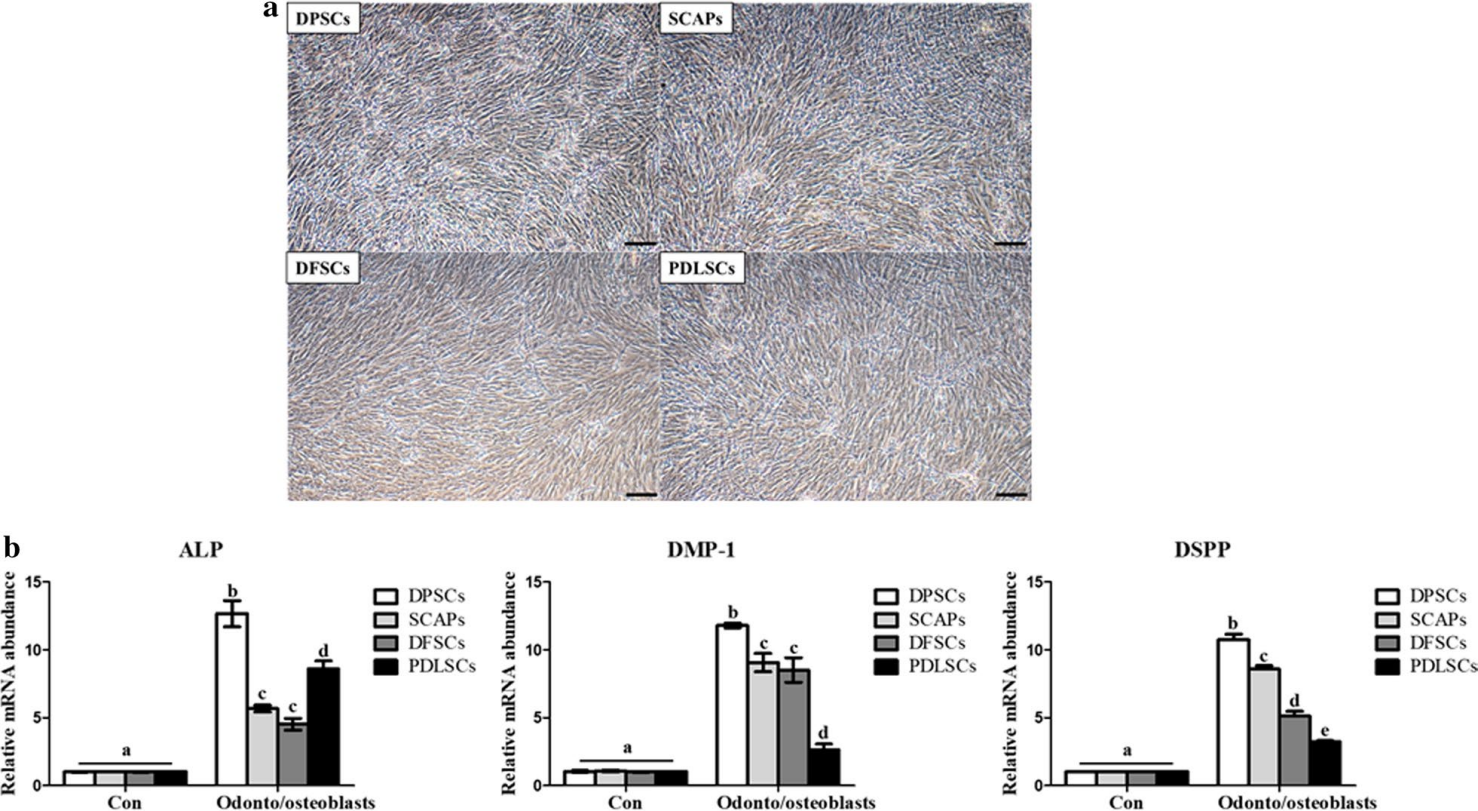
Cellular morphologies and odonto/osteoblast-specific gene expression levels of differentiated odonto/osteoblasts from four types of dental stem cells. **a** odonto/osteoblasts were induced from DPSCs, SCAP, DFSCs, and PDLSCs showed mineral matrix formation (scale bar = 100 µm). **b** Expression of odonto/osteoblast specific genes (*ALP, DMP-1*, and *DSPP*) was highest in odonto/osteoblasts from DPSCs. Data are represented by the mean ± SD of four independent experiments. Lettered subscripts indicate statistical differences between groups (*p* < 0.05)

All the DPSCs, SCAP, DFSCs, and PDLSCs were evaluated for their differentiation potential into odonto/osteogenic induction medium supplemented with 10% FBS, 1% penicillin/streptomycin, 10 mM ß-glycerophosphate, 50 µg/mL ascorbate-2-phosphate. The Mineralized matrix formation was observed in the cells. (Fig. 3a). Data demonstrated that the expression of *ALP, DMP-1*, and *DSPP* was highest (*p* < 0.05) in differentiated odonto/osteoblasts derived from DPSCs (Fig. 3b). PDLSCs displayed comparatively low expression for *DMP-1* and *DSPP* than others, whereas significantly high *ALP* expression was shown by PDLSCs than SCAPs and DFSCs. Both the SCAPs and DFSCs showed marginal significant differences for the expression of odonto/osteoblast specific markers.

**Fig. 4:**
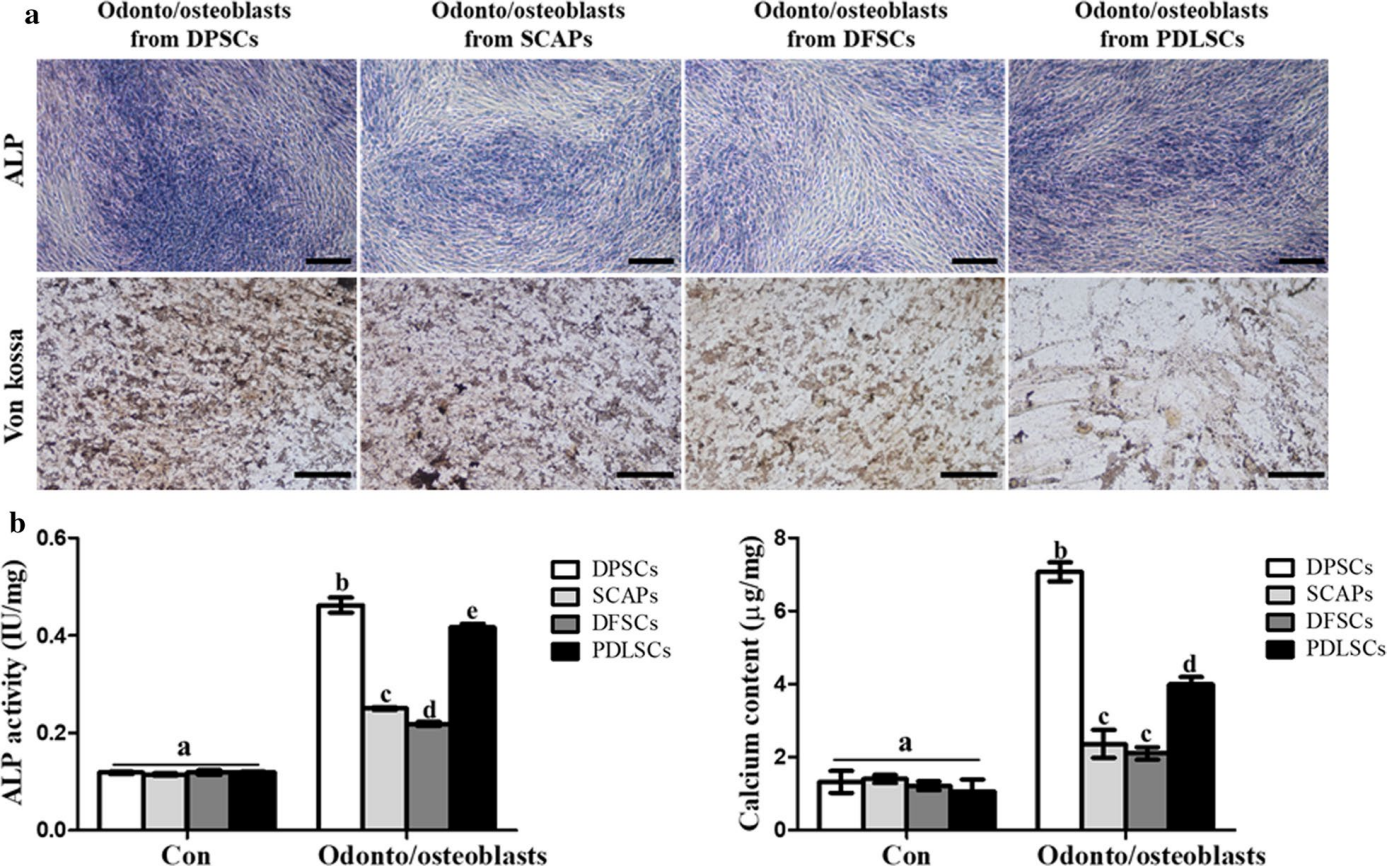
The evaluation of mineralization and calcium content in odonto/osteoblasts from four types of dental stem cells. **a** Odonto/osteoblasts from DPSCs had the strongest staining intensity using ALP and Von kossa stains (scale bar = 100 µm). **b** ALP activity and calcium content were highest in the odonto/osteoblasts from DPSCs. Data are represented by the mean ± SD of four independent experiments. Lettered subscripts indicate statistical differences between groups (*p* < 0.05)

**Fig. 5:**
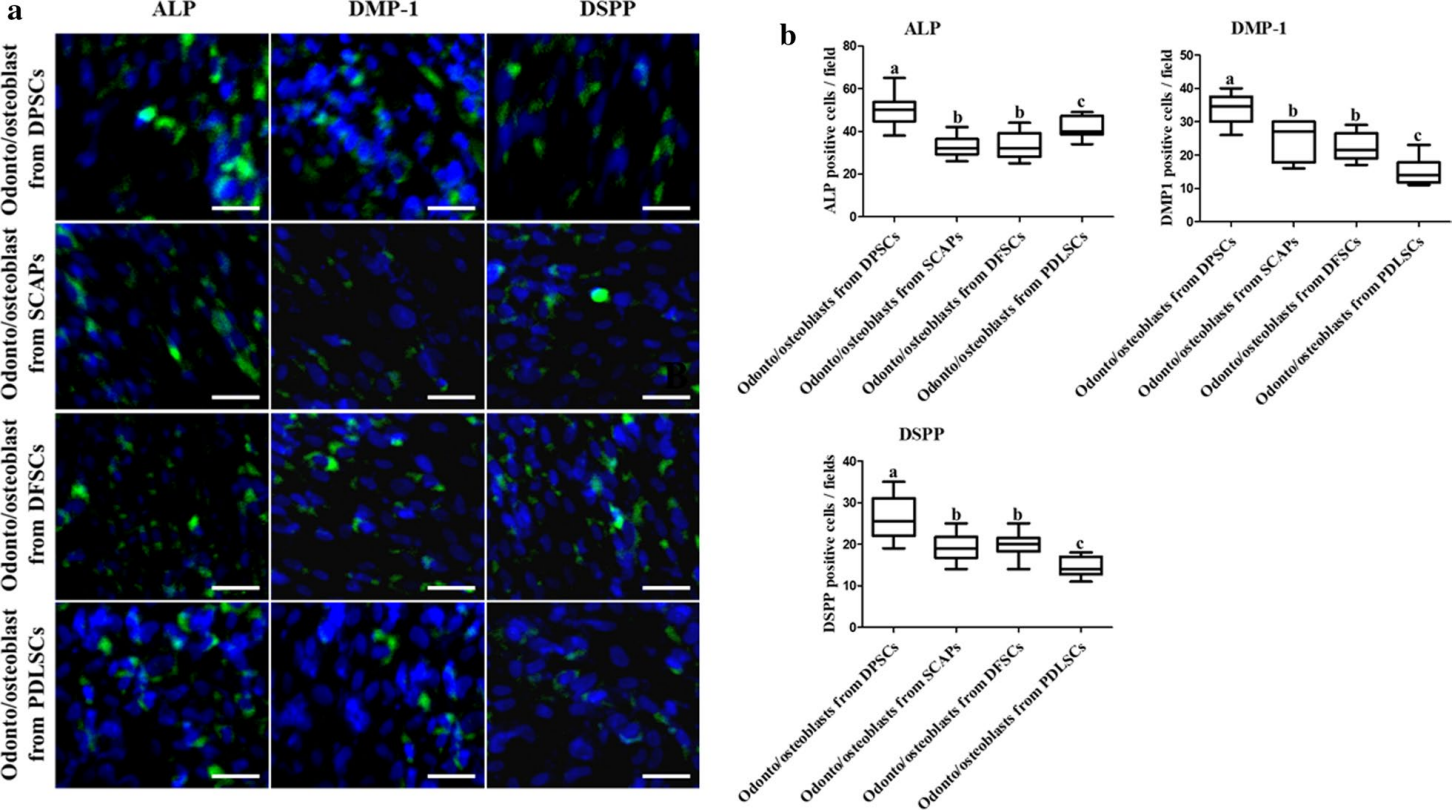
Quantified immunocytochemical analysis of odonto/osteoblasts. **a** Immunofluorescence staining revealed positive expression of odontoblast specific markers (ALP, DMP-1, and DSPP) in odonto/osteoblasts from all dental stem cells. Nuclei were stained with DAPI (scale bar 25 µm). **b** All marker proteins positive cells were showed in odonto/osteoblasts from DPSCs, indicating that DPSCs were the highest potential stem cell source for in vitro odontoblastic differentiation. Data are represented by the mean ± SD of four independent experiments. Lettered subscripts indicate statistical differences between groups (*p* < 0.05)

Von kossa and ALP staining visualized mineral nodules formation during odonto/osteoblast differentiation. Staining intensity was highest in odonto/osteoblasts from DPSCs 14 days after induction (Fig. 4a). ALP activity and calcium content were also highest (*p* < 0.05) in odonto/osteoblasts from DPSCs (Fig. 4b, c). Like RT-qPCR results, PDLSCs also demonstrated high ALP staining and ALP activity than SCAPs and DFSCs (Figs. 3b, 4a, b). Protein expression of ALP, DMP-1, and DSPP was evaluated by immunocytochemical staining. Dental-derived MSCs cultured in odonto/osteoblast differentiation medium (ODM) expressed odonto/osteoblast specific markers (Fig. 5a). After differentiation, odonto/osteoblasts derived from DPSCs showed highest (*p* < 0.05) number of positive cells (Fig. 5b). SCAPs, DFSCs and PDLSCs revealed the similar number of odonto/osteoblast specific markers positive cells as evaluated by RT-qPCR and immunocytochemical staining. Based on highest odonto/osteoblast differentiation ability by DPSCs among all other cell groups, DPSCs were selected for further experimentation.

### Supplementation with E2 promotes odonto/osteoblast differentiation

Cell proliferation demonstrated by PDT assay showed significantly decreased doubling time compared to non-E2 supplementation groups in all dental MSCs (Additional file 1: Fig. S1). The mRNA levels for the pro-apoptosis related genes (BAX and BAK) were significantly decreased and anti-apoptosis related genes (BCL2 and BIRC) were increased in cultured MSCs with E2 supplementation (Additional file 2: Fig. S2).

**Fig. 6:**
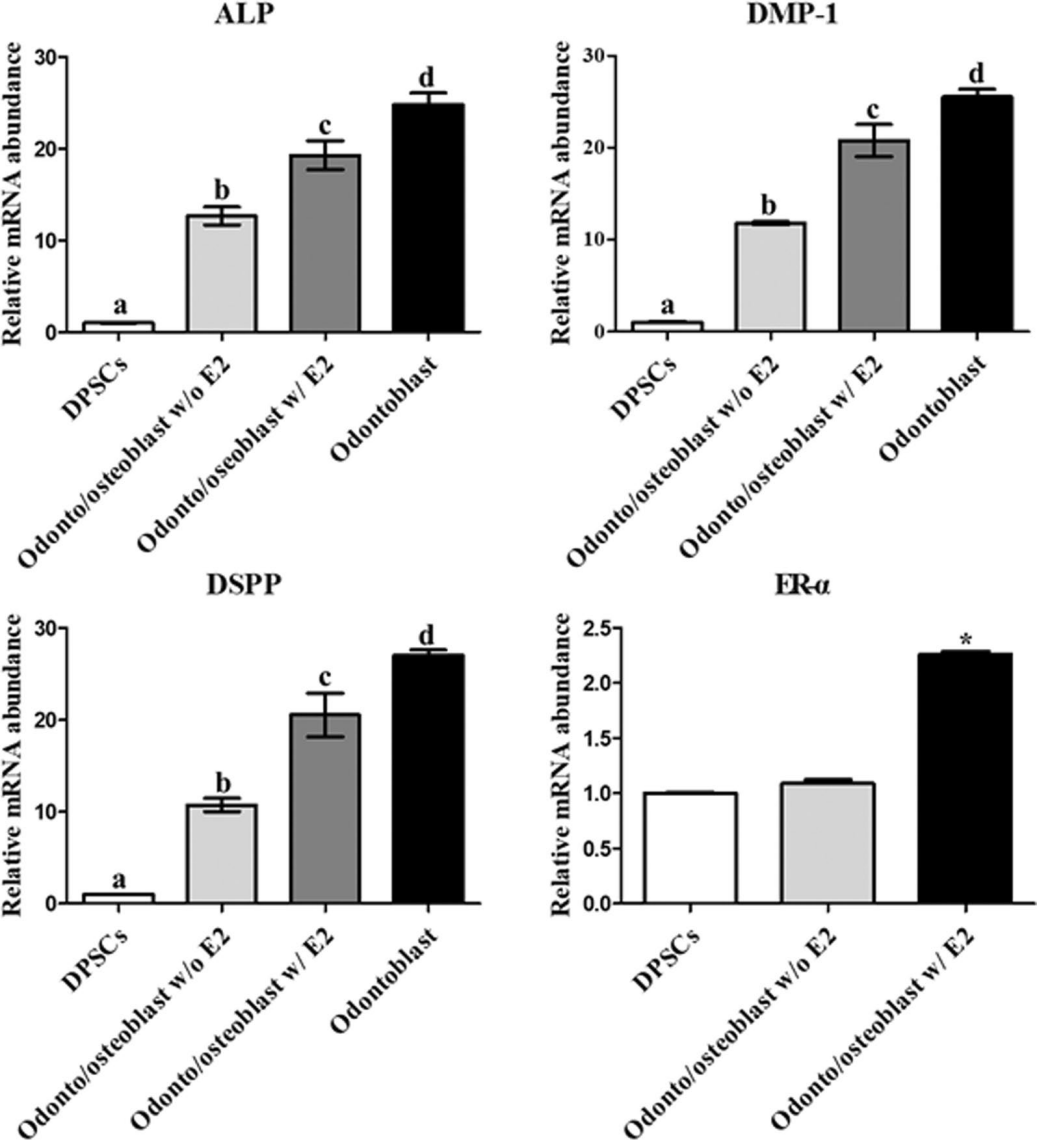
Analysis of E2 supplementation in odontoblastic differentiation of DPSCs. The mRNA levels of odontoblast specific markers *ALP, DMP-1*, and *DSPP* were highest in odonto/osteoblasts supplemented with E2. Levels were similar to those in cultured pure odontoblasts from dental chamber (Odontoblast). Expression of ER-α was highest in E2 supplemented differentiated cells. Data are represented by the mean ± SD of four independent experiments. Lettered subscripts indicate statistical differences between groups (*p* < 0.05)

**Fig. 7:**
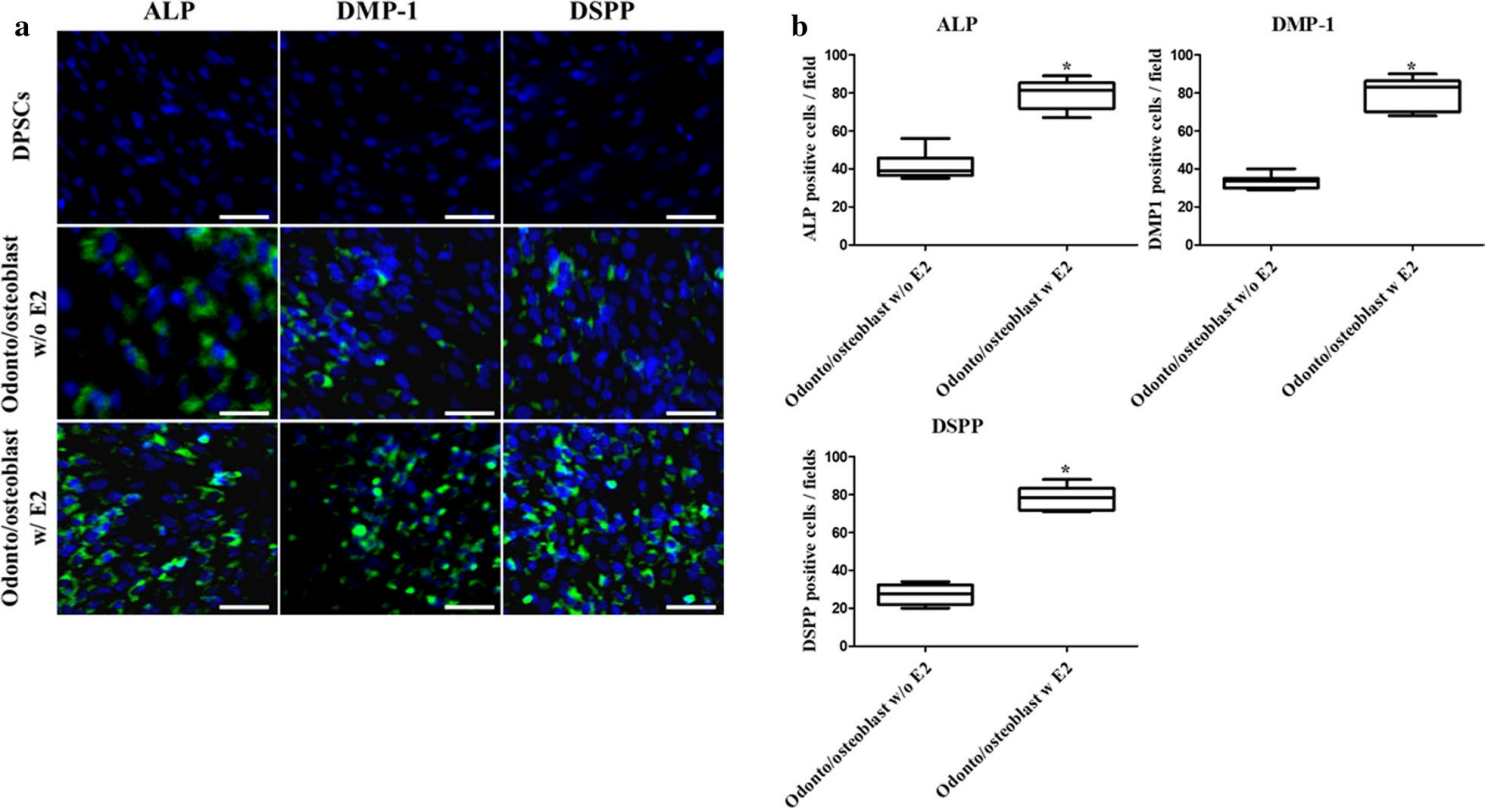
Quantified immunocytochemical analysis of odonto/osteoblasts according to E2 supplementation status. **a** Odonto/osteoblast specific proteins ALP, DMP-1, and DSPP were positively expressed in odonto/osteoblasts and non-differentiated DPSCs were negatively expressed. Nuclei were stained with DAPI (scale bar = 25 µm). **b** odonto/osteoblasts supplemented with E2 showed the highest number of ALP, DMP-1 and DSPP positive cells, indicating E2 supplementation enhanced odonto/osteoblast differentiation. Data are represented by the mean ± SD of four independent experiments. Lettered subscripts indicate statistical differences between groups (*p* < 0.05)

**Fig. 8:**
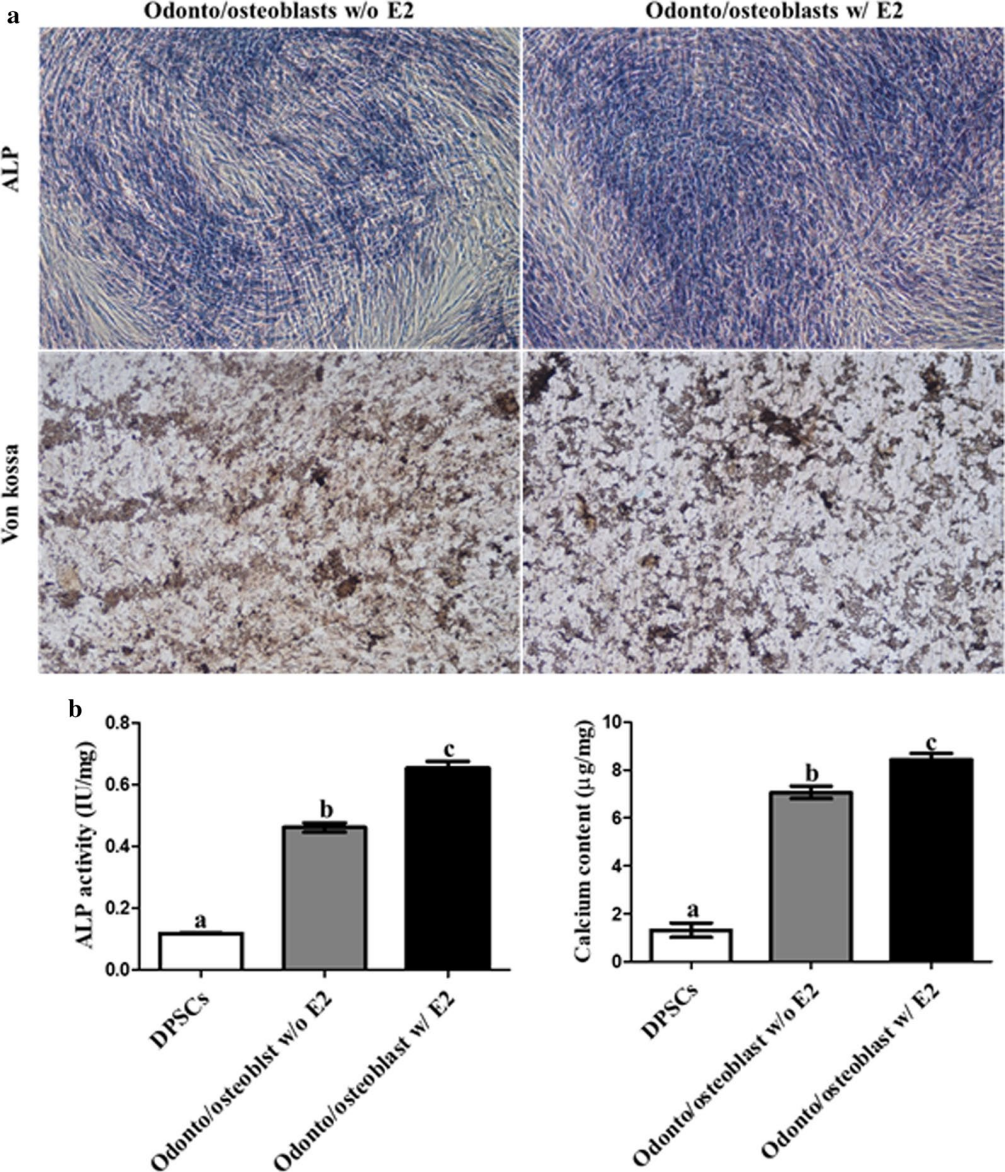
The evaluation of mineralization and calcium content in odonto/osteoblasts according to E2 supplementation status. **a** Odonto/osteoblasts w/ E2 had the strongest staining intensity using ALP and Von kossa stains (scale bar = 100 µm). **b** ALP activity and calcium content were highest in the odonto/osteoblasts w/E2. Data are represented by the mean ± SD of four independent experiments. Lettered subscripts indicate statistical differences between groups (*p* < 0.05)

Odonto/osteoblast-differentiated DPSCs were cultured in ODM with and without 10 µM E2 for 2 weeks to investigate the effect of E2 supplementation on odonto/osteoblast differentiation in DPSCs. After differentiation, the odonto/osteoblast specific markers ALP, DMP-1, and DSPP and ER-α were analyzed to determine the effect of E2 supplementation. The expression of genes (*ALP, DMP-1*, and *DSPP*) and *ER-α* was higher in differentiated odonto/osteoblasts supplemented with E2 than without E2 supplementation (*p* < 0.05) (Fig. 6). To determine the protein expression of odonto/osteoblast related markers, immunocytochemical staining was performed. ALP, DMP-1, and DSPP were not expressed in non-differentiated DPSCs (Fig. 7a). These proteins were expressed in odonto/osteoblasts regardless of E2 supplementation (Fig. 7a). Furthermore, odonto/osteoblasts supplemented with E2 showed significantly increased number of ALP, DMP-1 and DSPP positive cells as compared to odonto/osteoblasts without E2 supplementation (Fig. 7b). The ALP staining intensity, which represents mineralized extracellular matrix formation, was highest in cells induced with E2 supplementation (Fig. 8a). ALP activity and calcium content were significantly increased (*p* < 0.05) on supplementation with E2 during odonto/osteoblast differentiation (Fig. 8b).

## Discussion

The present study was carried out to establish an efficient method for differentiation of MSCs into odonto/osteoblasts. Therefore, we isolated DPSCs, SCAPs, DFSCs and PDLSCs from a same donor to compare their biological properties in order to select most efficient source for odonto/osteoblast differentiation and the effect of E2 supplementation for improved differentiation capacity was also further evaluated. It had been demonstrated that variations in dental stem cell differentiation potentials might be related to their origins. Dental MSCs from different sources vary in their odonto/osteoblast differentiation properties, even though they were isolated from same donor while expressing same MSC properties [19, 24]. This finding was extended by evaluating the characteristics of four kinds of dental MSCs from a same donor. The morphology, multi-lineage differentiation capacity, CD markers expression and cell proliferation did not differ among the cells suggesting that all dental MSCs were successfully isolated and shared similar stemness characteristics. The dental pulp is a terrific source of stem cells for odontoblast differentiation due to its distinct cellular composition [1, 10, 11, 15, 16, 25] and contains post- mitotic primary odontoblasts and undifferentiated DPSCs, which produce tertiary dentin to protect pulp tissue against external stimuli [26, 27]. When dental MSCs were differentiated into odonto/osteoblasts, the gene expression levels of odontoblast specific markers, including *ALP, DMP-1* and *DSPP* were significantly higher in differentiated cells as compared to non-differentiated cells. Moreover, the odonto/osteoblasts from DPSCs showed higher expression of odontoblast markers than others. On the other hand, DMP-1 and DSPP were comparatively low expressed in PDLSCs compared to others but their ALP expression was significantly higher than SCAPs and DFSCs. The basic mechanism behind varied expression for ALP, DMP-1 and DSPP by the induced PDLSCs is not known and need further elucidation.

It has been demonstrated that the dentin is composed of mineral substance and odontoblasts formed these mineral matrix and deposited calcium [28]. All odonto/osteoblasts from dental MSCs were positively stained with ALP and Von kossa. Furthermore, the odonto/osteoblasts from DPSCs were most strongly stained with both. Several studies have mentioned the ALP activity as an early marker of odontoblast differentiation [11, 28]. In our study, the expression of ALP activity was significantly higher expressed in odonto/osteoblasts when compared to undifferentiated dental MSCs. Especially, the expression of ALP activity was most significantly highly expressed in odonto/osteoblasts from DPSCs than other groups. The mineralization process was performed by transporting calcium ions in odontoblasts and the deposition of calcium indicated mineralization in odontoblast differentiation [29–31]. Induced DPSCs exhibited higher calcium content levels in comparison to other treated groups. When odonto/osteoblasts from all the four dental sources were evaluated for their immunocytochemical expression targeting specific markers, similar results were shown by DPSCs with higher number of positive cells in comparison to other differentiated groups. No significant differences were shown by differentiated SCAPs and DFSCs whereas lower number of positive cells were shown by PDLSCs. The possible reason behind these results could be due to the different origins and their varied response under used in vitro induction conditions. Recent studies revealed that odontoblasts and osteoblasts share similar origin and both cells had the capacity of mineral formation and calcium deposition [28]. Furthermore, it has also been demonstrated that DMP-1 and DSPP are highly expressed in odontoblasts than osteoblasts and ALP, which is also primary marker of osteoblasts showed different expression pattern with DMP-1 and DSPP [32]. Results from the present study showed higher expression level of odontoblast specific markers by differentiated DPSCs both at mRNA and protein levels. Which means they possess highest potency towards odonto/osteoblast differentiation.

Although many studies have been performed on the differentiation of MSCs into odonto/osteoblasts, however reports on culture conditions and methods for improving differentiation efficiency are still limited. Conditioned media from tooth germ and natural dentin matrix have been used for in vitro odonto/osteoblast differentiation from stem cells [6, 8, 9], however, it is difficult to maintain homogenous, constant conditions when using this media. In the present study, ODM contain various combinations of ingredients, including ß-glycerophosphate, ascorbate-2-phosphate, and dexamethasone, which makes odontoblast induction easier and produce constant results [1, 23]. Recent studies revealed that estrogen is essential for in vivo and in vitro odontogenic and osteogenic differentiation of DPSCs [16, 25, 33]. Additionally, several studies have also mentioned the association between periodontitis and autoimmune disease pathway and triggering factors [34–36]. The estrogen hormone has shown immunomodulation capacity and proven effective in periodontitis. Furthermore, E2 supplementation downregulated cellular apoptosis in MSCs, promoted cellular growth and proliferation, and helped in upregulating the odontoblast differentiation ability of the induced cells [15, 37]. Therefore, we conducted a study using E2 supplementation as a growth factor for efficient dental pulp regeneration. The abundant levels of ER-α and ER-ß were detected in the mRNA of human dental pulp stem cells by RT-PCR [16, 38]. E2 and ER-α had the most potent biological activities and played critical roles in osteogenic and odontogenic differentiation [15, 39]. E2 stimulated odonto/osteoblast differentiation of human dental cells, which was associated with ER-α, c-Src, and MAPK pathways [15]. Consistently, present study also demonstrated that E2 supplementation substantially increased in vitro odonto/osteoblast differentiation potential of DPSCs. In addition, ER-α was significantly higher in the E2 supplemented cell cultures, indicating ER-α mediated signaling might be related to E2-induced odonto/osteoblast differentiation, which were in accordance with the similar findings of previous studies [15, 36]. Moreover, the resident odontoblasts in the pulp chamber were harvested and cultured so that they can be used as a positive control [20]. Gene expression levels of odonto/osteoblast makers, including *ALP, DMP-1*, and *DSPP* were similar in resident odontoblasts and odonto/osteoblast from DPSCs, although slightly higher levels of *DMP-1* and *DSPP* were observed in the resident odontoblasts. This indicates that strategies employed for in vitro production of odonto/osteoblasts in the present study are efficient. The odonto/osteoblasts from DPSCs had similar characteristics to resident odontoblasts found in the dental pulp.

## Conclusion

From the present study, it has been concluded that dental pulp-derived MSCs had greater odonto/osteoblast differentiation potential than MSCs from other dental tissues, including dental follicle, apical papilla, and periodontal ligament. Moreover, E2 supplementation can significantly enhance the in vitro odonto/osteoblast differentiation potential. Additionally, in vitro induced odonto/osteoblasts are promising dental pulp regeneration agents in restorative dentistry that may become widely available, although further in vivo studies are needed.

## Supplementary information

**Additional file 1: Fig. S1.** Analysis of cell proliferation by population of doubling time (PDT). All dental MSCs showed significantly decreased doubling time compared to non-17ß-estradiol supplementation added group. Data are represented by the mean ± SD of four independent experiments. Lettered subscripts indicate statistical differences between groups (*p* < 0.05).

**Additional file 2: Fig. S2.** Apoptosis-related gene expression levels of four kinds of dental MSCs according to E2 supplementation. Cultured dental MSCs with E2 supplementation showed significantly decreased pro-apoptosis related genes (BAX and BAK) expression level and increased anti-apoptosis related genes (BLC2, BIRC). There was no difference during the culturing of cells. The values were expressed as mean ± SD of three independent experiments (*p* < 0.05).

## Data Availability

All data used and/or analyzed during the current study available from the corresponding authors on reasonable request.

## Abbreviations

MSCs: Mesenchymal stem cells
DPSCs: Dental pulp stem cells
SCAPs: Stem cells from apical papilla
DFSCs: Dental follicle stem cells
PDLSCs: Periodontal ligament stem cell
CD: Cluster of differentiation (classification determinant)
E2: 17ß-estradiol
ALP: Alkaline phosphatase
DSPP: Dentin sialophosphoprotein
DMP-1: Dentin matrix acidic phosphoprotein 1
ER-α: Estrogen receptor-α
MAPKs: Mitogen-activated protein kinases
DPBS: Dulbecco’s phosphate-buffered saline
ADMEM: Advanced Dulbecco’s modified Eagle’s medium
FBS: Fetal bovine serum
FITC: Fluorescein isothiocyanate
BSA: Bovine serum albumin
cDNA: Complementary DNA
YWHAZ: Tyrosine 3-monooxygenase/tryptophan 5-monooxygenase activation protein: zeta polypeptide
DAPI: 4′,6-Diamidino-2-phenylindole
ODM: Odonto/osteoblast differentiation medium
