# Supplementary figures and images for: Evaluation of odonto/osteogenic differentiation potential from different regions derived dental tissue stem cells and effect of 17β-estradiol on efficiency
Source: BMC Oral Health. 2021 Jan 7;21:15. doi: 10.1186/s12903-020-01366-2 (PMC7792121; doi:10.1186/s12903-020-01366-2)

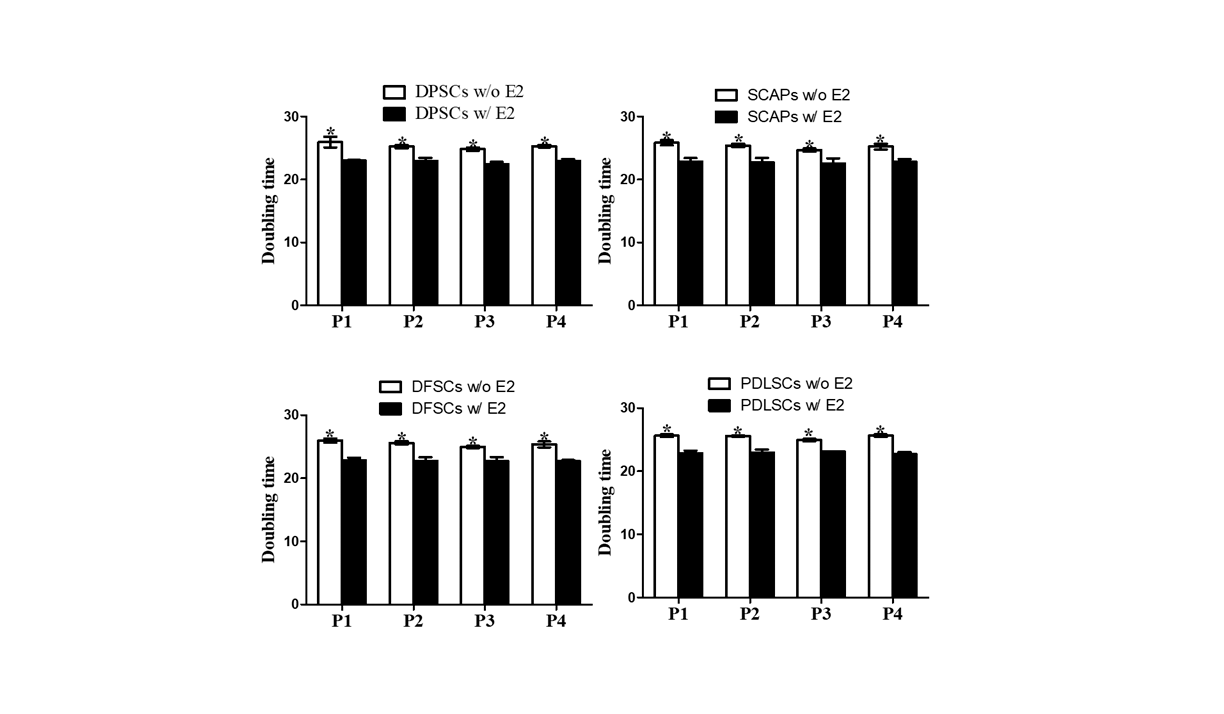

Supplement: Supplementary file 1 — Additional file 1: Fig. S1. Analysis of cell proliferation by population of doubling time (PDT). All dental MSCs showed significantly decreased doubling time compared to non-17ß-estradiol supplementation added group. Data are represented by the mean ± SD of four independent experiments. Lettered subscripts indicate statistical differences between groups (p < 0.05). [file 12903_2020_1366_MOESM1_ESM.tif]

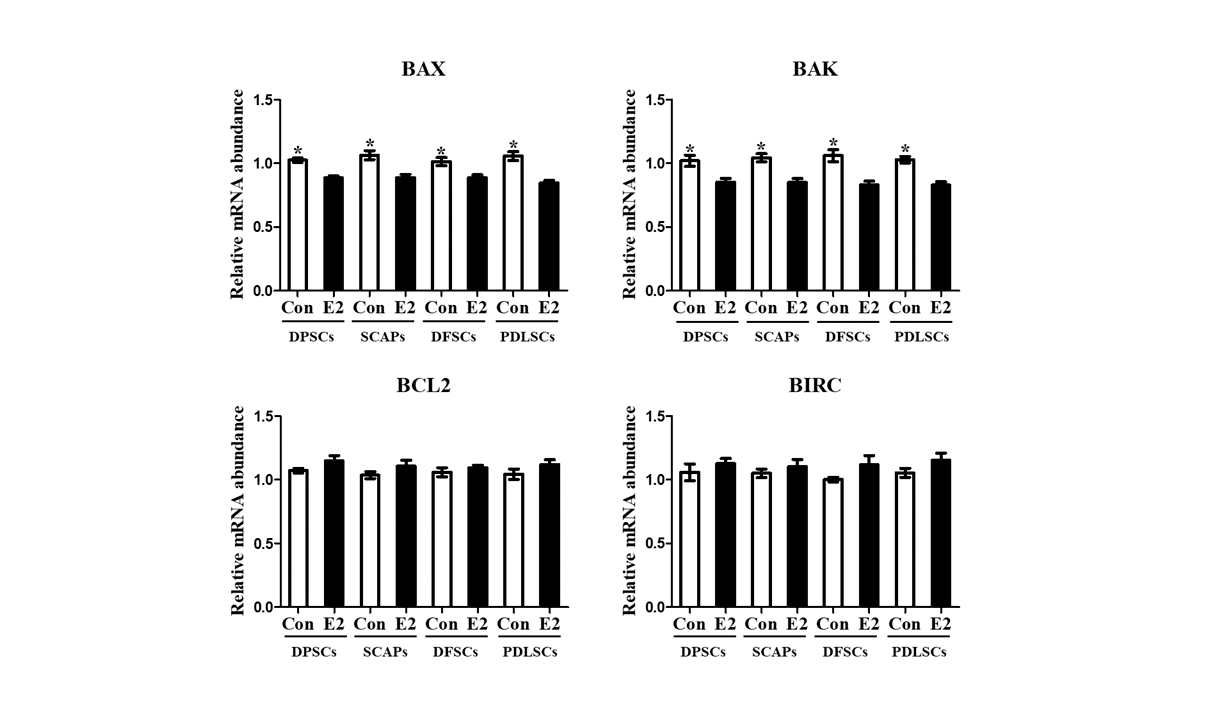

Supplement: Supplementary file 2 — Additional file 2: Fig. S2. Apoptosis-related gene expression levels of four kinds of dental MSCs according to E2 supplementation. Cultured dental MSCs with E2 supplementation showed significantly decreased pro-apoptosis related genes (BAX and BAK) expression level and increased anti-apoptosis related genes (BLC2, BIRC). There was no difference during the culturing of cells. The values were expressed as mean ± SD of three independent experiments (p < 0.05). [file 12903_2020_1366_MOESM2_ESM.tif]
